# Drinking Water Quality: Taking the Lead and Copper Rule to Task

**Published:** 2006-05

**Authors:** Scott Fields

When testing in 2003 found higher-than-allowable lead levels in the District of Columbia’s drinking water, it hit awfully close to home for some law makers, prompting three U.S. legislators to direct the Government Accountability Office (GAO) to evaluate how well the EPA regulates lead levels in drinking water. In January 2006, after a year-long investigation, the GAO reported that although the EPA says lead levels in drinking water systems have dropped since the early 1990s, the agency in fact has no data—which states are supposed to provide—to support that finding for about 30% of medium and large municipal systems. Additionally, although the EPA requires states to report on lead-in-water “milestones,” or measures that must be met, the agency lacks those data for 72% of water systems.

The report centered around the question of how well the agency enforces its 1991 Lead and Copper Rule. This rule requires water utilities to sample lead levels in homes and, at certain trigger points, to notify customers and sometimes take remedial action.

The Lead and Copper Rule is unusually tricky to enforce, because the contaminants in question are out of the control of the water utilities. “What makes lead so unique is that it’s picked up in the distribution system; everything else, like *E. coli*, is treated at the water treatment facility,” says John Stephenson, director of natural resources and environment at the GAO.

Usually lead is introduced to drinking water in the service lines, which connect individual buildings to main water lines. These service lines are often owned by individuals rather than utilities. Lead may also be introduced within the house itself, from lead pipes or solder that connects copper pipes in the house. Because lead enters drinking water so late in the pipeline, samples must be taken from the taps of individual structures rather than from a central distribution point. Typically, building owners are asked to provide these samples.

The Lead and Copper Rule stipulates that in the largest systems—50,000 or more users—only 100 homes have to be tested, says Stephenson. Generally speaking, testing is done every three years. “We didn’t get into the reasonableness of the samples, but it isn’t a very large sample in the first place,” he says. “It’s not until more than ten percent of those tests are above acceptable levels that you have to do anything about it.”

That was the case in Washington, where 40,000 water service lines were replaced after the District of Columbia Water and Sewer Authority found drinking water lead above the action level of 15 parts per billion in 73% of the 4,613 homes tested. All of the homes tested had lead service lines.

One reason the EPA was short on data may have been that some states decided to concentrate their scant resources on lead management rather than lead reporting, says Steve Via, a regulatory engineer for the nonprofit American Water Works Association, whose membership is drawn from water utilities. “Would you rather see a state with limited resources spending a lot of time managing the data up the chain so that somebody can have a relatively simple time compiling a report? Or would you rather see them put the money into having their people in the field helping people who have problems either complying or trying to do a better job?” he asks.

An appendix to the report notes that the agency continued to assess penalties during the period in question. As for the future, the EPA has developed a plan to improve its enforcement of the rule, and is preparing revisions that will address some of the issues raised in the GAO report, says Veronica Blette, a special assistant to the director of the EPA Office of Ground Water and Drinking Water. The agency must also notify Congress as to how it will address the GAO’s recommendations, and will periodically report on its progress.

For now, Stephenson says, the GAO has no further role to play in the process. “It’s really up to the Congress to keep oversight pressure on the GAO to stay involved, to ask us again to look at it—and they may down the road.”

## Figures and Tables

**Figure:**
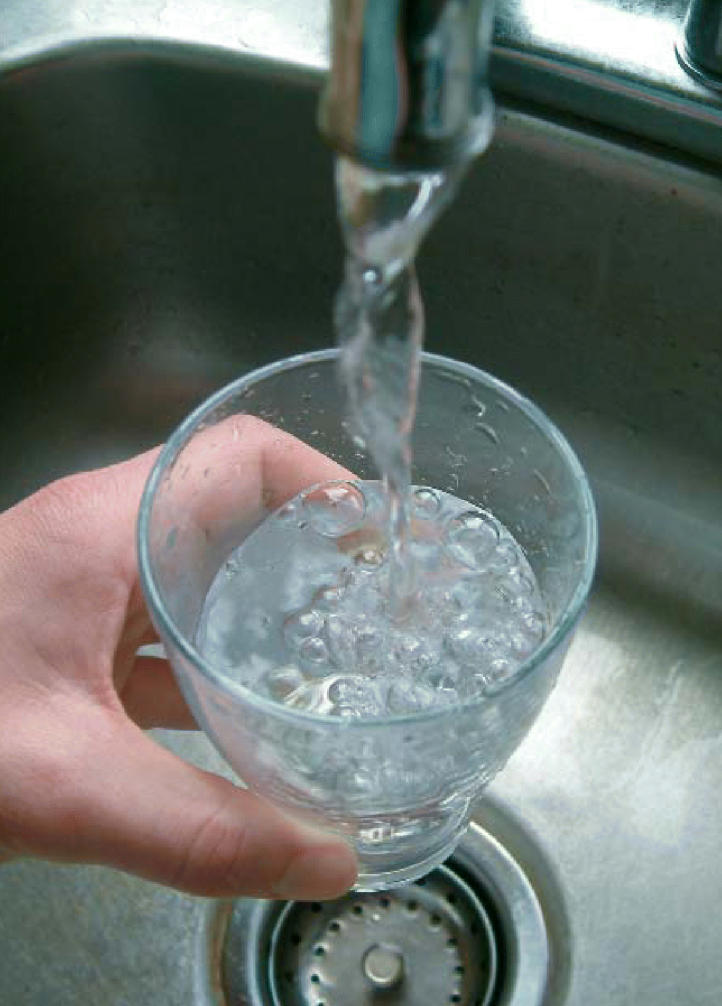
Cloudy on the details. A GAO report on the EPA Lead and Copper Rule shows that enforcement is not a clear-cut outcome.

